# Feasibility of images acquired using smartphone camera for marginal and internal fit of fixed dental prosthesis: comparison and correlation study

**DOI:** 10.1038/s41598-024-55711-4

**Published:** 2024-03-04

**Authors:** Young-Tak Son, KeunBaDa Son, Gyeong-o Eo, Kyu-Bok Lee

**Affiliations:** 1 Department of Dental Science, Graduate School, Kyungpook National University, Daegu, Republic of Korea; 2 Advanced Dental Device Development Institute, Kyungpook National University, 2177 Dalgubuldaero, Jung-Gu, Daegu, 41940 Republic of Korea; 3 Department of Smart Software, Yonam Institute of Technology, Jinju-Si, Gyeongsangnam-Do Republic of Korea; 4 Department of Prosthodontics, School of Dentistry, Kyungpook National University, Daegu, Republic of Korea

**Keywords:** Smartphone camera, Optical microscope, Intraclass correlation coefficients, Marginal and internal fit, Health care, Medical research

## Abstract

This study aimed to measure marginal and internal fit using images captured with both an optical microscope and a smartphone camera, comparing the fit measurement performance of these devices and analyzing their correlation. Working casts (with 10 posterior and 10 anterior teeth) created to fabricate fixed dental prostheses were used. These working casts were scanned using a desktop scanner (E1) to design an interim crown, and the designed interim crown was fabricated using a three-dimensional (3D) printer. Utilizing the silicone replica technique, the fabricated interim crown replicated the fit, which was then captured using both an optical microscope and a smartphone camera. The captured images were used to measure the marginal and internal fit according to the imaging device. Intraclass correlation coefficients (ICC) were used for reliability analysis according to the imaging device. Furthermore, the Wilcoxon signed-rank test was adopted for the comparative evaluation of the marginal and internal fit between the imaging devices (α = 0.05). The measurement results of the marginal and internal fit according to the optical microscope and smartphone camera did exhibit a significant difference (*P* < 0.05). The ICC between the two devices showed an “excellent” agreement of over 0.9 at all measurement points (*P* < 0.001). A smartphone camera could be used to obtain images for evaluating the marginal and internal fit.

## Introduction

When manufacturing dental prostheses, it is important to ensure the proper formation of the marginal and internal fit, which represents the gap between the prepared teeth and the intaglio surface of the prosthesis^1,2^. An incorrect formation may cause bacterial plaque accumulation, cement dissolution, gingival inflammation, hypersensitivity, secondary caries, and finally clinical failure of the prosthesis^3–5^. Previous studies have reported that the clinically acceptable range of marginal fit for prosthesis is 120 µm^6,7^. Therefore, the marginal and internal fit has been evaluated using various methods in many previous studies to ensure accurate prosthesis fabrication.

Various methods are used to evaluate the marginal and internal fit, such as the silicone replica technique^6^, superimposition after 3D scan^8,9^, weight technique^10^, microcomputed tomography^11,12^, optical coherence tomography^4,13^, and cutting and measurement after cementation of the prosthesis^14,15^. The silicone replica technique has been used in several studies as a gold standard for marginal and internal fit evaluation owing to its simplicity and excellent accuracy in a nondestructive method^16–18^. This technique involves the injection of silicone into the intaglio surface of the prosthesis and evaluation of the marginal and internal fit. However, its disadvantages are the need to use professional measurement equipment (optical microscope) to evaluate the marginal and internal fit and the long measurement time^19^. For this reason, the silicone replica technique has been used only in research, not in clinical situations. Therefore, a simple and accurate method for marginal and internal fit evaluation without the use of professional measurement equipment is needed.

Optical microscope is used in many fields, such as material science, biology, and dentistry^3,20,21^. However, it requires a separate purchase of equipment for measurement and professional training for use^22,23^, limiting its application in clinical situations and making quantitative evaluation of prostheses difficult. With the recent upgrade of smartphones, smartphone cameras have also improved, making it easy to obtain high-resolution images^24,25^. Furthermore, the processing speed, storage space, and computing power of smartphones have improved, making them convenient for users. Several previous studies used smartphone cameras as alternatives to optical microscopes^24–28^. Some researchers also obtained high-magnification images by attaching a lens to a smartphone or using additional equipment^25,27,28^. In addition, previous studies compared the usability of surgical microscopes and smartphones in clinical practice without attachments to smartphones^24,26^. At present, smartphones are widely used by clinical experts and researchers^29^. These devices do not require additional startup costs and increase user accessibility^30,31^. To acquire images using an optical microscope and a smartphone camera, and to assess reliability by evaluating the marginal and internal fit, additional research is warranted.

In previous studies, to compare the accuracy of an optical microscope and a smartphone camera, researchers either used a cradle to fix the smartphone or manufactured and used a special device^20,23,25,26,28,30–32^. These methods were used to obtain consistent smartphone camera images. However, special devices incur additional fabrication costs and have lower accessibility, thus reducing the advantage of smartphone cameras, which are convenient and incur little additional cost^33–35^. Furthermore, the results of research using specially designed equipment could only be verified under the same conditions, and they are difficult to apply to general clinical conditions. Therefore, it is necessary to verify without applying special devices to compare the images obtained using an optical microscope and smartphone camera.

This study aimed to compare the measurement performance and evaluate the correlation between the two devices when measuring marginal and internal fit through images acquired using an optical microscope and a smartphone camera. The null hypothesis is that there is no significant difference between the two devices in the evaluation of marginal and internal fit using images obtained through an optical microscope and a smartphone camera, and that the two devices exhibit a significant correlation.

## Methods

### Procedure for methods

**Figure 1:**
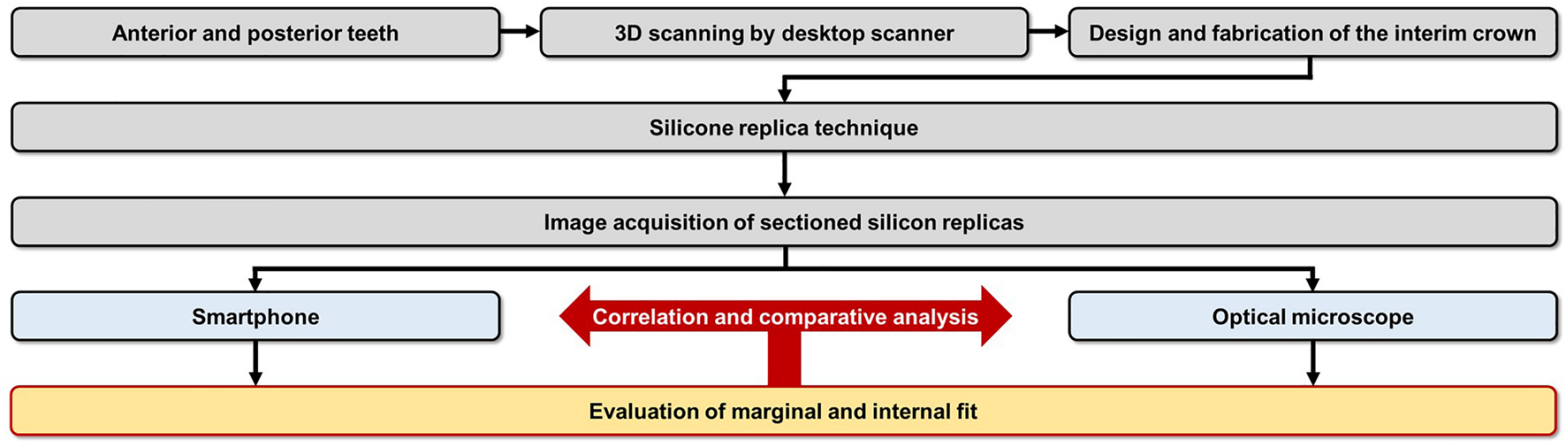
Experimental design.

This study was conducted using working casts created to fabricate fixed dental prostheses stored at Kyungpook National University Dental Hospital. Posterior and anterior working casts were scanned to obtain virtual working casts. Interim crowns designed based on the virtual working casts were printed. The silicone replica technique was employed to measure the marginal and internal fit of the fabricated interim crowns. The replica was captured using an optical microscope (IMS 1080P, Sometech Inc., Seoul, Republic of Korea) and smartphone camera (iPhone 12, Apple, Inc., Cupertino, CA, USA), and the images obtained were compared and evaluated for marginal and internal fit using an image processing software (NIH ImageJ; NIH, Bethesda, MD) (Fig. 1).

### Fabrication of working casts and silicone replica to measure marginal and internal fit

To determine the required number of samples per group, five pilot experiments were conducted prior to the present study. A total of 20 samples per group was determined based on the following results using a power analysis software (G*Power v3.1.9.2; Heinrich-Heine-Universität, Düsseldorf, Germany): actual power, 80.7%; power, 80%; and α, 0.05.

**Figure 2:**
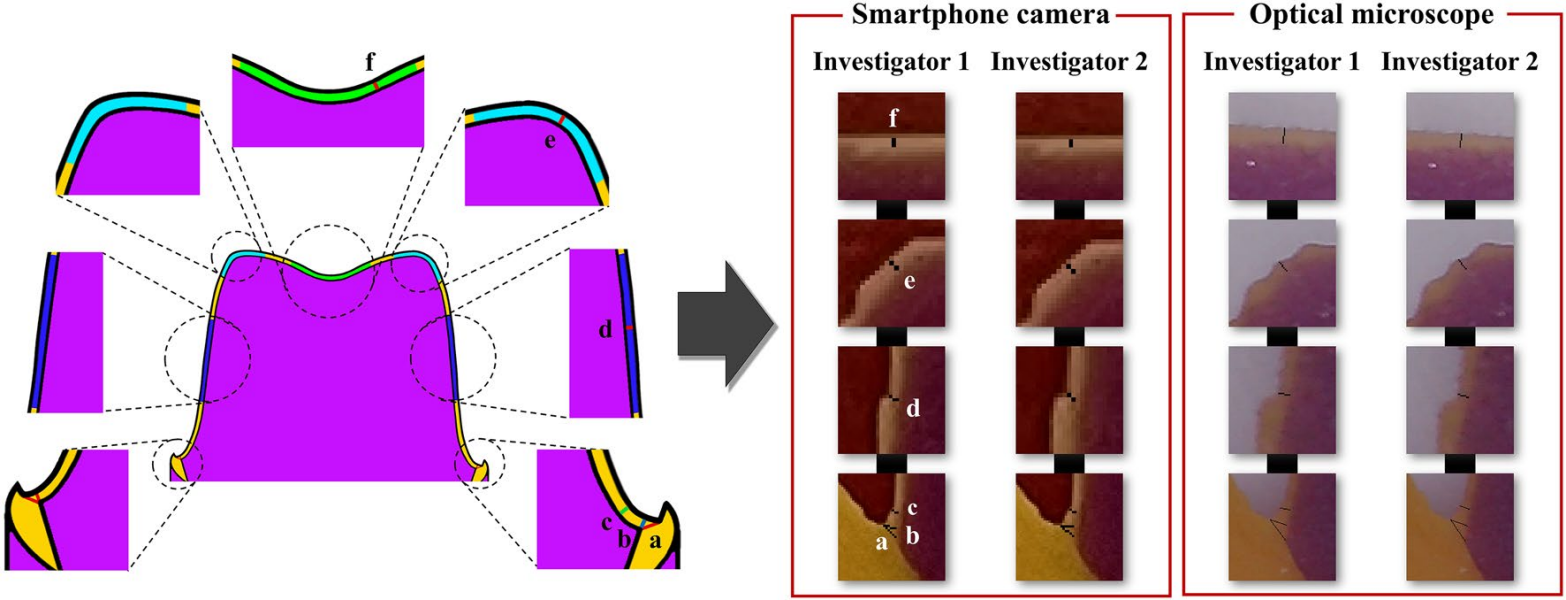
Comparison of the evaluated internal and marginal fit position and the smartphone and optical microscope image. (**a**) Absolute marginal discrepancy. (**b**) Marginal gap. (**c**) Chamfer gap. (**d**) Axial gap. (**e**) Angle gap. (**f**) Occlusal gap.

A total of 20 working casts created to fabricate fixed dental prostheses, which were stored in the Department of Prosthodontics of Kyungpook National University Dental Hospital, were selected. Working casts were selected with natural abutments present, regardless of anterior or posterior teeth, for the fabrication of dental fixed prostheses such as crowns (10 anterior and 10 posterior teeth). In this experiment, cases involving implants with metal abutments or instances where the working cast was broken or damaged, affecting the abutment, were excluded. Using a desktop optical scanner (E1; 3Shape, Copenhagen, Denmark), all working casts were obtained as virtual working casts, which were saved as standard tessellation language (STL) files. Before use, the desktop optical scanner was calibrated according to the manufacturer’s protocols. For the virtual working casts, a dental CAD software (3Shape Dental System; 3Shape, Copenhagen, Denmark) was used to design interim crowns according to the abutments of each virtual working cast. For the internal space setting of the interim crown, a 60-µm cement space and a 1-mm distance to the finish line were applied. The designed interim crowns were printed with a dedicated photocurable resin (RAYDENT C&B; Ray, Seoul, Republic of Korea) using a 3D printer (Meg-printer 2; MegaGen, Daegu, Republic of Korea). The print was postphotopolymerized according to the manufacturer’s protocols. The silicone replica technique was employed for the marginal and internal fit replication of the fabricated interim crown. After filling the inner surface of the interim crown with a silicone indicator (Aquasil Ultra Monophase; Dentsply Detrey GmbH, Konstanz, Germany) and placing the interim crown on the abutment, continuous force was applied until polymerization was completed. Subsequently, a silicone with a different color from the replicated silicone was filled in, polymerized, and then separated. To obtain a slide-shaped cut surface, the replicated silicone was cut twice in the direction of the buccolingual plane based on the center of the occlusal surface. Cut silicone replicas were used to measure the marginal and internal fit (Fig. 2).

### Image acquisition using optical microscope and smartphone camera

**Figure 3:**
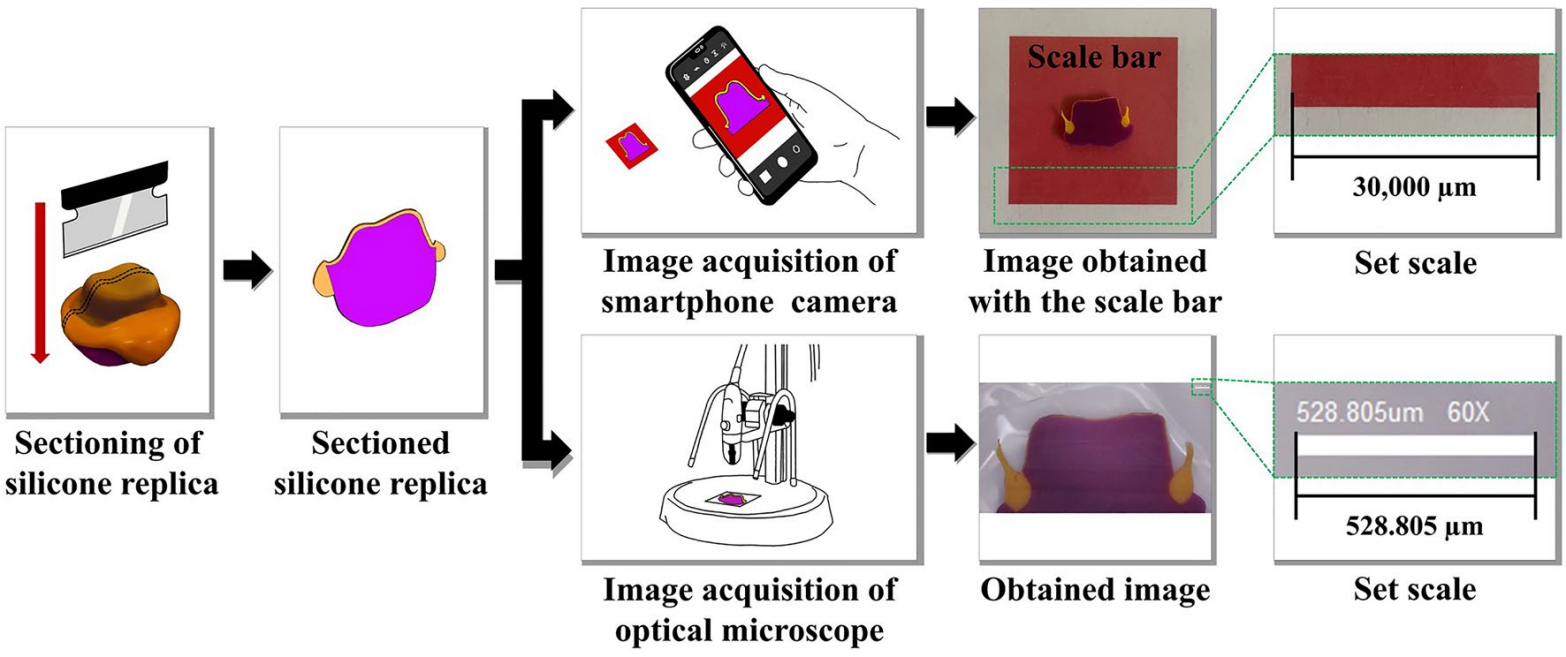
Procedure for image acquisition and set scale.

To acquire images for the measurement of the marginal and internal fit, iPhone 12 pro and IMS 1080P were used. The acquisition was performed by one experienced investigator (Y.-.T.S.). All images obtained using a smartphone camera were captured in room light. Images obtained using a smartphone camera did not provide a scale bar. Therefore, a standard scale bar was formed in the background so that the scale bar could be obtained in the image when shooting with a smartphone camera. The scale bar was designed as a red square with a side of 30,000 µm and was printed using an A4 paper-based desktop inkjet printer. Sectioned silicone replica was positioned at the center of the printed scale bar and captured with a smartphone camera (Fig. 3). A camera application installed on the smartphone was used by default without magnification. During the capture, the silicon specimen was placed at the center of the grid in the smartphone screen and captured vertically using the level, a function of the application. The procedure was performed by the investigator with the smartphone in hand, and images were captured at the shortest distance between the specimen and the camera that could be focused on the specimen. All smartphone images were saved in JPEG format, 3024 × 4160 pixel dimension, and 72 dpi resolution. After capturing the images using the smartphone camera, they were captured again using an optical microscope (IMS 1080P; SOMETECH) at a magnification of × 60. The specimen was positioned so that all measurement points could be captured in one image. The image captured for marginal and internal fit measurement was checked in a dedicated software (ITPlus 5.0, SOMETECH, Seoul, Korea) of the optical microscope, and the image was exported.

### Measurement of the marginal and internal fit

Marginal gap (MG) and absolute marginal discrepancy (AMD) were evaluated as the measuring points of marginal fit. The internal fit measurement points were evaluated for chamfer gap, axial gap, angle gap, and occlusal gap (Fig. 2). The captured images were evaluated for the marginal and internal fit using an image processing software. After loading the image within the image processing software, the ‘Set scale’ function was used to set the actual length per pixel of the image. The scale of the photos obtained via the smartphone camera was set based on the scale bar of 30,000 µm in the image (Fig. 3). On the other hand, the image captured using the optical microscope was set-scaled based on the scale bar of 528.805 µm given in the dedicated software (Fig. 3). After setting the scale, the straight function was used to display the fit of the measurement point and measure the distance. Evaluation of the marginal and internal fit was performed by two dental science specialists to verify the reliability of the data.

### Statistical analysis

All data were analyzed using the SPSS statistical software (release 25.0; IBM Corp, Chicago, IL, USA). The reliability between the two investigators was confirmed via Intraclass correlation coefficient (ICC) for the marginal and internal fit measurement results for both images obtained using the smartphone camera and optical microscope methods and the ICC was used to confirm the reliability of the marginal and internal fit measurement results according to the device used. ICC values less than 0.5, between 0.5 and 0.75, between 0.75 and 0.9, and greater than 0.9 indicated poor, moderate, good, and excellent reliability, respectively^36^. The Wilcoxon signed-rank test was employed to confirm the difference according to the device used to capture the images (optical microscope and smartphone camera). To compare the differences between the optical microscope and smartphone camera groups using the calculated mean and standard deviation (SD), the effect size of Cohen's d was calculated using the following formula:

$${{\text{Cohen}}}^{\mathrm{^{\prime}}}\mathrm{s d}= \frac{{Mean}_{1}-{Mean}_{2}}{\sqrt{\frac{({n}_{1}-1){{SD}_{1}}^{2}+({n}_{2}-1){{SD}_{2}}^{2}}{{n}_{1}+{n}_{2}-2}}}$$

The calculated effect size was classified as small (0.2), medium (0.5), and large (d ≥ 0.8).

## Results

Table 1 evaluates the interrater reliability of the marginal and internal fit measurement results of images acquired through a smartphone camera and optical microscope. The ICC value was 1.000 at all marginal and internal fit measurement points of the posterior and anterior teeth evaluated by two investigators, which indicates “excellent” interrater reliability (*P* < 0.001).

**Table 1:** Interrater reliability comparison results for marginal and internal fit measurement results of images obtained via smartphone camera and optical microscope methods.

Tooth type	Marginal and internal fit	Intraclass correlation coefficient for interrater reliability	P*
Posterior teeth	AMD	1.000	< 0.001*
	MG	1.000	< 0.001*
	Chamfer	1.000	< 0.001*
	Angle	1.000	< 0.001*
	Axial	1.000	< 0.001*
	Occlusal	1.000	< 0.001*
Anterior teeth	AMD	1.000	< 0.001*
	MG	1.000	< 0.001*
	Chamfer	1.000	< 0.001*
	Axial	1.000	< 0.001*
	Incisal	1.000	< 0.001*

*Significance for comparing interrater reliability determined by the intraclass correlation coefficient, *P* < 0.05. 1.000 represents perfect agreement, and lower numbers represent lower agreement.

*AMD* absolute marginal discrepancy, *MG* marginal gap.

**Figure 4:**
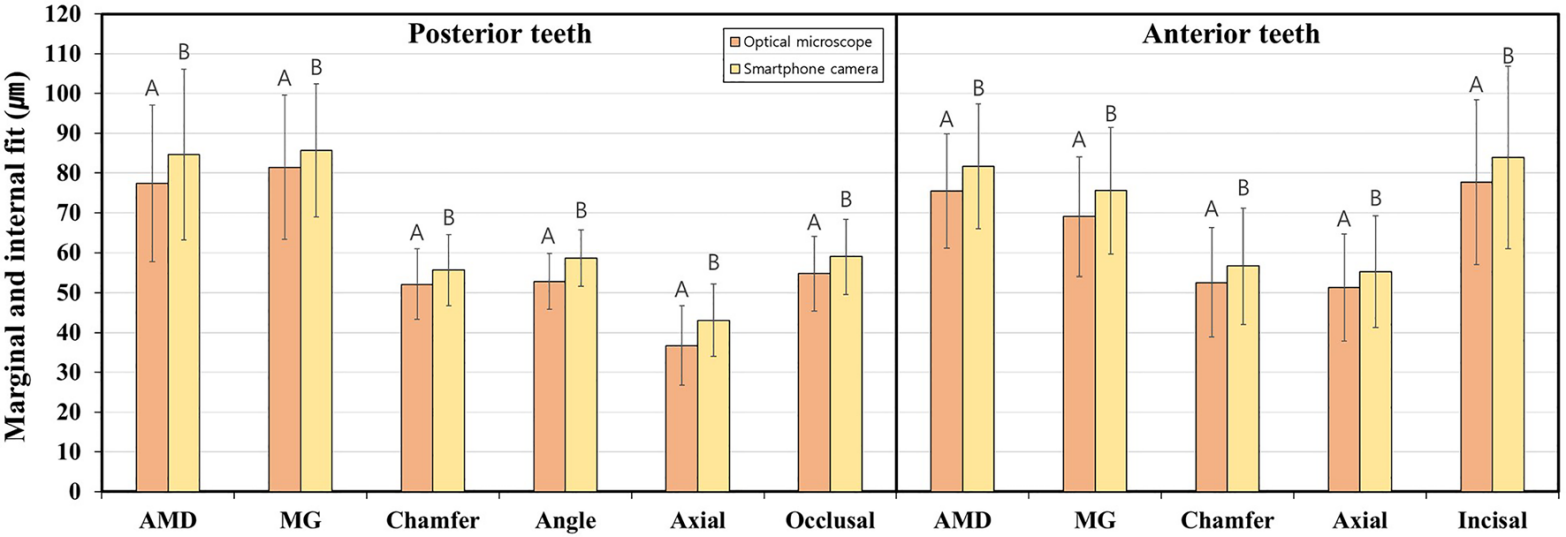
Comparison of the marginal and internal fit of the posterior and anterior teeth using images acquired using an optical microscope and smartphone camera. Identical letters indicate that difference between the groups is not significant (*P* ≥ 0.05).

Table 2 and Fig. 4 analyze the comparison and correlation of the two image devices by measuring the marginal and internal fit of images obtained using a smartphone camera and an optical microscope. Significant differences were observed in all marginal and internal fit measurement points of the posterior and anterior teeth evaluated in images obtained using the optical microscope and smartphone camera (*P* < 0.05). However, when the ICC values between the optical microscope and the smartphone camera were compared, all measurement points of the marginal and internal fit had ICC values exceeding 0.9, indicating “excellent” reliability (*P* < 0.001). The effect size between the optical microscope and smartphone camera showed a small value of less than 0.2 for AMD, MG, and chamfer in the case of posterior teeth. Also, it showed a medium but relatively small value of less than 0.5 for angle, axial, and occlusal. In the case of anterior teeth, chamfer, axial, and incisal showed small values of less than 0.2, and AMD and MG showed medium but relatively small values of less than 0.5.

**Table 2:** Comparison and correlation analysis of the two imaging devices by measuring the marginal and internal fit of images obtained using a smartphone camera and an optical microscope.

Tooth type	Marginal and internal fit	Device	Mean	SD	95% Confidential interval		P*	Intraclass correlation coefficient of two devices	P**	Effect size^†^
					Lower	Upper				
Posterior teeth	AMD	Optical microscope	77.39	39.34	64.81	89.98	< 0.001*	0.982	< 0.001**	0.18
		Smartphone camera	84.63	42.95	70.90	98.37				
	MG	Optical microscope	81.44	36.21	69.86	93.02	< 0.001*	0.958	< 0.001**	0.12
		Smartphone camera	85.69	33.54	74.96	96.41				
	Chamfer	Optical microscope	52.15	17.56	46.53	57.77	0.005*	0.946	< 0.001**	0.2
		Smartphone camera	55.61	17.86	49.90	61.32				
	Angle	Optical microscope	52.86	13.87	48.43	57.30	< 0.001*	0.902	< 0.001**	0.42
		Smartphone camera	58.65	13.97	54.18	63.12				
	Axial	Optical microscope	36.74	19.90	30.37	43.10	< 0.001*	0.946	< 0.001**	0.33
		Smartphone camera	43.11	18.09	37.33	48.90				
	Occlusal	Optical microscope	54.74	18.59	48.80	60.69	0.004*	0.925	< 0.001**	0.23
		Smartphone camera	58.98	18.73	52.99	64.97				
Anterior teeth	AMD	Optical microscope	75.42	28.68	66.25	84.59	< 0.001*	0.976	< 0.001**	0.21
		Smartphone camera	81.69	31.24	71.70	91.68				
	MG	Optical microscope	69.07	30.06	59.46	78.69	< 0.001*	0.977	< 0.001**	0.21
		Smartphone camera	75.55	31.84	65.37	85.74				
	Chamfer	Optical microscope	52.59	27.36	43.84	61.34	< 0.001*	0.989	< 0.001**	0.14
		Smartphone camera	56.62	29.25	47.27	65.98				
	Axial	Optical microscope	51.30	26.88	42.70	59.89	< 0.001*	0.991	< 0.001**	0.14
		Smartphone camera	55.26	27.99	46.31	64.21				
	Incisal	Optical microscope	77.67	41.43	58.28	97.06	< 0.001*	0.989	< 0.001**	0.14
		Smartphone camera	83.93	45.82	62.49	105.38				

*AMD* absolute marginal discrepancy, *MG* marginal gap.

*Significance for comparing optical microscope and smartphone camera determined by the Wilcoxon signed-rank test, *P* < 0.05. In the intraclass correlation coefficient, 1.000 represents perfect agreement between the two devices, and lower numbers represent lower agreement.

**Significance for comparing optical microscope and smartphone camera determined by intraclass correlation coefficient, *P* < 0.05.

^†^Cohen’s d was used as the effect size.

## Discussion

In the present study, the reliability of the obtained images was verified and evaluated by investigating the correlation between the two devices when measuring the marginal and internal fit. Images were captured using both an optical microscope and a smartphone camera. Significant differences were observed in the marginal and internal fit measurement results according to the device used (*P* < 0.05; Table 2). However, the ICC values of the two imaging devices exceeded 0.9, indicating “excellent” reliability (*P* < 0.001; Table 2). Therefore, the null hypothesis was partially accepted.

The selection of appropriate evaluation methods and devices is essential to ensure the quality of research. It is a common task to evaluate the performance of a new method or device in terms of reliability by comparing it with commonly used ones^36^. Reliability refers to how the results of compared methods or devices are consistently reproduced^37^. ICC is commonly recommended as a measure for assessing the reliability of experimental methods^38^. In previous studies, ICC was used to evaluate reliability between evaluation methods. Koo et al.^39^ introduced the basic concept of reliability analysis. Based on the 95% confidence interval of the ICC estimate, values less than 0.5, between 0.5 and 0.75, between 0.75 and 0.9, and greater than 0.9 indicated poor, moderate, good, and excellent reliability, respectively. Correlation refers to the relationship between phenomena or objects, beyond methods or devices. In other words, it is a statistical method that explains the degree to which two or more variables cooperate^40^. Banik et al.^25^ reviewed recent trends in smartphone-based detection for biomedical applications and reported correlations with optical microscopes and smartphone cameras. Furthermore, Talebian et al.^28^ reported an “excellent” correlation when they compared the use of a smartphone camera and observation using a manual-based optical microscope. The previously mentioned studies also conducted comparisons between smartphone cameras and optical microscopes, but it is challenging to compare them with the method used in this study due to differences in the intended use of the evaluated smartphones and the units of measurement. However, in this study, the marginal and internal fit were evaluated on images captured using an optical microscope and smartphone camera, and reliability between the imaging devices was evaluated using ICC. As a result of this study, the ICC values of the marginal and internal fit evaluated on images obtained using an optical microscope and smartphone camera exceeded 0.9 at all measurement points. This result suggests that the optical microscope and smartphone camera used did not affect the marginal and internal fit measurement results. When comparing the difference between an optical microscope and a smartphone camera using Cohen’s d effect size, all values indicated an effect size close to small. These results showed that there was no difference between the two devices when measuring marginal and internal fit using an optical microscope and a smartphone camera. Furthermore, when the optical microscope and smartphone camera were compared, no significant difference was observed in all marginal internal fit of the posterior and anterior teeth. Therefore, this result indicated that a smartphone camera could be used instead of an optical microscope when evaluating the marginal and internal fit within the limitations of this study. However, according to the results of this study, there was a statistically significant difference between the optical microscope and the smartphone camera. The smartphone camera's measured value was relatively high, so the marginal and internal fit obtained with the two devices were not completely identical. In all data, the measurement value of the smartphone camera was higher than that of the optical microscope. This could be explained by the difference in the distance per pixel of the image due to the variation in the resolution between the optical microscope and smartphone camera. In this study, the horizontal length of 1 pixel of the image of the smartphone camera was about 24.9 µm, whereas the image of the optical microscope was about 4.4 µm of the horizontal length of 1 pixel. As the size of 1 pixel of the image increases, the boundary between the teeth area and the fit area becomes difficult to recognize, resulting in low accuracy in gap measurement (Fig. 2). As the length that can be obtained at 1 pixel increases, it becomes more challenging to discern the starting point distinguished by different colors. This is because, within the length of 1 pixel, only one color information can be represented when there is a boundary area, making it difficult to find the starting point of the boundary. In this study, due to the smartphone camera resolution of 24.9 µm per pixel, there was a limitation that, if a boundary area existed within this measurement range, the color information was limited, and skilled operators had to rely on intuition to find it. In such cases, a limitation arose where measurement deviation increased. Furthermore, microscopes capable of representing a boundary area with a length of 4.4 µm per pixel showed relatively lower fit values compared to smartphone cameras, owing to their greater color information for depicting boundary regions. This limitation can be reduced in the future through efforts such as training artificial intelligence to establish consistent criteria when measuring distinct color boundaries. In previous studies, various methods were attempted to enhance the accuracy of smartphone camera photos^28,41,42^. When the camera shakes while shooting by hand, issues such as a decrease in image quality and the inability to clearly recognize boundaries occur. To overcome this, in other studies, researchers used a holder that could stabilize the camera for capturing images^28^. As mentioned earlier, there is a limit to the length that 1 pixel can represent. To tackle such challenges, other studies have equipped smartphone cameras with special lenses capable of enhancing photo magnification^41,42^. When a magnifying lens is attached to a smartphone camera, it allows for capturing more information per pixel regarding length. However, in this study, photos were obtained solely through the use of the mobile phone camera without the need for additional equipment. The reason for not using additional equipment in this study was to replicate the clinical situation accurately, as the usability may decrease when additional equipment is employed in a clinical setting. According to previous research, it is possible to increase resolution through image upsampling using deep learning^43,44^. In this study, we exclusively used smartphone cameras without any additional equipment; however, in the future, applying image upsampling may enable obtaining higher-accuracy images without the need for other devices. Therefore, the accuracy of the fit images obtained using smartphone cameras with applied image upsampling should also be assessed in the future. A slight error according to the imaging device may cause the fit measured in µm to be out of the clinically acceptable range (< 120 µm). Previous studies have suggested that care should be taken when fabricating prostheses, as lesions may occur if the fit is out of the clinically acceptable range^1–5^. Therefore, additional research is still warranted to improve the resolution of smartphone images to make the fit measurement results similar between smartphone cameras and optical microscopes.

In most previous studies, special devices were manufactured and used to overcome the limitations of smartphones. Talebian et al.^28^ attached a lens to a smartphone camera to enlarge the sample and designed and 3D-printed a holder to fix the smartphone. Ayardulabi et al.^41^ reviewed various methods of attaching devices to use a smartphone as a microscope. In addition, Zhu et al.^42^ designed and manufactured a smartphone epifluorescence microscope and attached it to the smartphone camera to verify its performance. However, in the present study, no additional device was attached to the smartphone, and images were captured without magnification. Nevertheless, this study proved that it is possible to use a smartphone instead of an optical microscope when measuring the marginal and internal fit. These results can improve accessibility in dental clinical situations where quantitative measurement of the marginal and internal fit is not performed due to the need to use professional equipment and the additional equipment purchase costs. Furthermore, additional costs can be prevented by using smartphones, which are owned by most clinical experts. Therefore, methods that can be adopted using only a smartphone without an attached equipment need to be developed.

In the present study, an image processing software, ImageJ, was used to measure the marginal and internal fit of images obtained using a smartphone camera and optical microscope. Son et al.^45^ demonstrated a difference in the accuracy of the dental scanner used depending on the software program using 3D analysis. Schmalzl et al.^46^ reported that the accuracy of intraoral scanners was affected by software updates. Furthermore, Fortin et al.^47^ mentioned that differences in the software used could cause variations in the measurement results; they also compared the reliability difference between ImageJ and OsiriX. The present study did not use a dedicated optical microscope measurement software to eliminate errors in the measurement accuracy according to the software. The usability of ImageJ has been proven in several previous studies, and this software is used as a standard tool^47–49^. Therefore, in the present study, images obtained using the optical microscope and smartphone camera were evaluated using ImageJ, and errors caused by the software were eliminated.

This study has several limitations that need to be acknowledged. Because the results were evaluated for one type of optical microscope and smartphone camera, further research on various types of imaging devices is warranted. Because the smartphone was held by the investigator’s hand when capturing images, the accuracy should be compared and verified with the smartphone in a fixed state. Additional verification for various dental clinical cases should be performed.

## Conclusion

Within the limitations of this in vitro study, the following conclusions can be drawn.

1. The optical microscope and smartphone camera used in this study affect the measurement results of the marginal and internal fit, but the reliability between the devices was excellent.
2. Therefore, within the limitations of the present study, a smartphone camera can be used instead of an optical microscope to obtain images for the marginal and internal fit evaluation.

## Data Availability

The datasets used and analyzed during the current study are available from the corresponding author on reasonable request.
